# HYpofractionated, dose-redistributed RAdiotherapy (HYDRA) versus conventional radiotherapy for head and neck cancer: planned interim analysis and dosimetric comparison from the phase I HYDRA trial

**DOI:** 10.1016/j.ctro.2026.101113

**Published:** 2026-01-25

**Authors:** Pascal A. Gunsch, Michiel Kroesen, Reno Debets, Stijn Keereweer, Esther van Meerten, Jaap Zindler, Erik van Werkhoven, Mischa Hoogeman, Gerda M. Verduijn, Remi A. Nout, Joris B.W. Elbers

**Affiliations:** a Department of Radiotherapy, Erasmus MC Cancer Institute, Rotterdam, the Netherlands; b Department of Radiotherapy, HollandPTC, Delft, the Netherlands; c Department of Medical Oncology, Laboratory of Tumor Immunology, Erasmus MC Cancer Institute, Rotterdam, the Netherlands; d Department of Otorhinolaryngology, Head & Neck Surgery, Erasmus MC Cancer Institute, Rotterdam, the Netherlands; e Department of Medical Oncology, Erasmus MC Cancer Institute, Rotterdam, the Netherlands; f Department of Radiotherapy, Haaglanden Medical Center, The Hague, the Netherlands

## Abstract

**Purpose:**

(Chemo)radiotherapy for squamous cell carcinoma of the oropharynx, hypopharynx, and larynx results in significant treatment burden and may cause radiation-induced lymphopenia (RIL), which is associated with worse survival. We aim to reduce treatment burden and RIL using HYpofractionation and Dose-redistribution in patients treated with proton or photon RAdiotherapy (HYDRA, NCT05364411).

**Methods:**

Patients receiving curative (chemo)radiotherapy for cT1-4 N0-3bM0 oropharyngeal and hypopharyngeal carcinomas are eligible. Referral for proton therapy is determined by model-based selection (MBS) according to Dutch protocols, resulting in a HYDRA-proton and HYDRA-photon cohort. HYDRA in 20 fractions constitutes 40 Gy to the elective volume, 55 Gy to gross tumour volume (GTV) + 5 mm and 59 Gy to GTV − 3 mm. Safety interim analyses are conducted in each cohort when ten patients complete 6 months follow-up. The interim results determine trial continuation and expansion to inclusion of laryngeal carcinomas according to predefined dose limiting toxicities (DLT). Per included HYDRA-patient, we perform an intra-patient plan comparison of the HYDRA plan versus the conventional treatment plan that was used for MBS.

**Results:**

The HYDRA-photon interim analysis (reached in March 2024, *n* = 10) showed one DLT (osteoradionecrosis), with no other late grade ≥ 3 toxicity after a median follow-up of 10.6 months. Among all enrolled HYDRA-patients (photons: *n* = 14; protons: *n* = 5), intra-patient plan comparison showed that HYDRA delivered a focal boost of Dmean 59.6 Gy (range 59.0–60.1 Gy) to the GTV, while on average, organs at risk received a reduced dose (HYDRA-photons: 0.4–4.5 Gy EQD2; HYDRA-protons: 2.1–5.0 GyE EQD2).

**Conclusions:**

The predefined interim analysis of HYDRA-photons showed one DLT. Per protocol, inclusion of laryngeal carcinomas in the photon cohort is now possible. The 20 fraction HYDRA schedule delivers a focal boost to the tumour with reduced dose to all organs at risk.

## Highlights

• HYDRA investigates hypofractionated (20 fractions), dose-redistributed (focal boost, lower elective dose) proton or photon therapy for HNSCC.
• The planned interim analysis of HYDRA photon therapy showed toxicity within predefined limits, enabling inclusion of laryngeal carcinoma.
• Acute toxicity of HYDRA is manageable and transient, with no tube feeding or opioid use beyond three months after treatment.
• Intra-patient dosimetric comparison shows reduced dose to organs at risk with HYDRA, mean focal boost to GTV centers was 59.6 Gy.

## Introduction

Head and neck squamous cell carcinoma (HNSCC) is the sixth most common cancer worldwide [1]. Definitive radiotherapy (RT) with or without concurrent chemotherapy is standard of care (SOC) as organ-sparing treatment for HNSCC of the oropharynx, hypopharynx, and larynx. Unfortunately, outcome following chemoradiotherapy (CRT) is relatively poor with overall survival rates of only 40% for locally advanced disease [2]. Additionally, radiotherapy to the head and neck region commonly results in severe toxicity with a significant impact on quality of life. It is therefore of great importance to improve survival rates and reduce therapy-related side effects.

While there is proven survival benefit of immune checkpoint blockade in metastatic HNSCC [3], [4], the promising combination of definitive chemoradiotherapy with PD-L1 inhibition did not result in a survival benefit [5], [6]. The failure of the combination of these treatments might be explained by the immunosuppressive effects of elective lymph node irradiation and the lymphotoxic effects of large-field fractionated radiotherapy, leading to radiation-induced lymphopenia (RIL) [7], [8], [9], [10], [11]. RIL is associated with decreased survival in HNSCC, as well as in other tumour types [12]. We hypothesize that by using fewer fractions (HYpofractionation), reducing the elective dose while increasing the dose to the tumour (Dose-redistribution) and by RAdiotherapy with protons instead of photons (HYDRA), both the immunosuppressive effects and treatment burden of radiotherapy can be reduced [13]. Ultimately, the purpose of HYDRA is to reduce treatment toxicity and to explore the possibility of integrated radiotherapy with immunotherapy.

Over the last decades, hypofractionated schedules have been adopted in a variety of other cancers, thus far leaving behind HNSCC [14]. Hypofractionation is historically considered too toxic in HNSCC, particularly considering late normal tissue damage. However, by the integration of novel concepts and previous work by others, we propose that it has now become technically and radiobiologically feasible. First, the elective field dose can be reduced by the combination of advanced multimodality imaging [15] and has been proven equally effective [16], [17]. Second, radiation volumes can be reduced without impairment of local control by state-of-the-art image-guided radiotherapy and dose delivery [18]. With these radiotherapy advancements, it has also become technically feasible to redistribute the radiation dose towards a lower dose on the outside of the target volume, while increasing the dose per fraction inside the tumour [19]. Recent radiobiological modelling by Shuryak et al. suggests that hypofractionation for HNSCC may result in higher tumour control with less radiation-induced toxicity, by more efficiently targeting accelerated repopulation [20], [21]. In addition, the α/β-value of HNSCC may be lower than previously assumed [22]. Several small trials have shown promising results of hypofractionated regimens in HNSCC [23], [24].

Although our ultimate goal is to integrate HYDRA with immunotherapy, the first objective is to determine the safety of HYDRA-photons and HYDRA-protons in two ongoing, parallel phase I trials. Within the current paper, we report on the predefined interim safety analysis of the first ten patients who received HYDRA-photon radiotherapy and perform an intra-patient treatment plan comparison between HYDRA and standard of care treatment plans for all patients included up to the interim analysis.

## Methods

### Study design

**Fig. 1:**
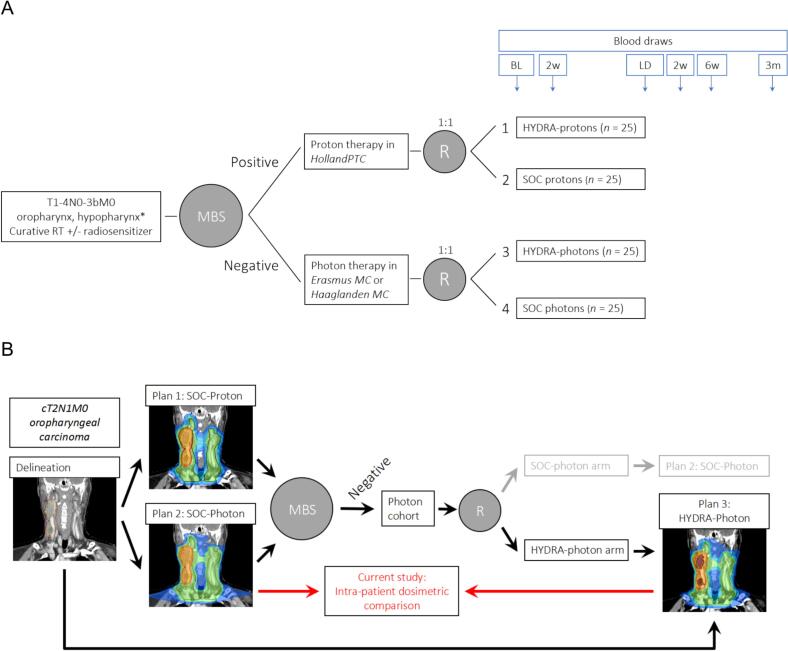
(a) HYDRA trial design and (b) an example of the study workflow for a single patient, showing that for HYDRA patients, three different treatment plans are created. * Laryngeal carcinomas are initially excluded until the planned safety interim analysis. Abbreviations: BL: baseline. LD: last day of treatment. SOC: standard of care. HNSCC: head and neck squamous cell carcinoma. MBS: model-based selection for proton therapy, according to Dutch selection criteria. R: randomization by minimization stratified by HPV status (positive / negative / unknown), tumour stage (I-II / III-IV), and concurrent radiosensitization (yes / no).

A detailed protocol of the ongoing multicentre Phase I HYDRA trial (NCT05364411) has been published previously [25]. A schematic overview is shown in Fig. 1.

In short, patients with cT1-4N0-3bM0 oropharyngeal and hypopharyngeal squamous cell carcinoma receiving definitive radiotherapy with curative intent (with or without concurrent cisplatin, carboplatin, or cetuximab) are eligible. Following Dutch guidelines, patients can be referred for proton therapy if they have model-based clinically relevant toxicity (NTCP) reductions [26]. To compare intra-patient NTCP reductions using model-based selection (MBS), clinical proton and photon therapy treatment plans are both created. After MBS, patients in the proton or photon therapy cohort are randomized for translational research purposes to either HYDRA or standard of care fractionation (Fig. 1). Blood will be collected for longitudinal immune profiling. For those patients who are allotted to the HYDRA treatment, a new treatment plan is created according to the HYDRA dose prescriptions.

### Treatment

All delineations are performed according to international contouring guidelines for the primary tumour [27] and the neck [27], [28]. The gross tumour volume (GTV) is determined by the combination of clinical examination, PET-CT or PET-MRI, and ultrasound. The HYDRA dose prescriptions are delivered in 20 daily fractions. SOC prescriptions are delivered in 35 fractions, 5 or 6 fractions per week.

#### Conventional clinical target volumes (CTV): CTV_4000/CTV_5425 and CTV_5500/CTV_7000

An elective dose of 40 Gy (HYDRA, CTV_4000) is prescribed to the GTV + 10 mm and elective lymph nodes, which corresponds to the standard of care CTV_5425. A boost of 55 Gy (HYDRA, CTV_5500) is prescribed to the GTV + 5 mm, which corresponds to the standard of care CTV_7000.

#### HYDRA focal boost target volume: GTV_5900

For HYDRA, a focal inhomogeneous boost of 59 Gy is prescribed to the primary tumour and the pathological lymph nodes (GTV_5900 = GTV − 3 mm). The mean dose of 59 Gy corresponds to an equal late normal tissue toxicity probability after conventionally fractionated radiotherapy of 70 Gy in 35 fractions, considering α/β = 3 Gy for late normal tissue complications.

The GTV_5900 is created using a 3 mm contraction margin to ensure that the 59 Gy boost always lies within the GTV (analogous to a negative PTV, to counter daily set-up errors). If, after this step, there is no GTV_5900 vol left in case of a small tumour, only the CTV_4000 and the CTV_5500 boost will be prescribed.

#### PTV expansion or robust optimization

For photon therapy, a planning target volume (PTV) expansion margin of 3 mm is applied in accordance with standard of care for the CTV_5500/CTV_7000 and the CTV_4000/CTV_5425 target volumes. As described above, the GTV_5900 is created using a 3 mm contraction margin analogous to a negative PTV. Thus, GTV_5900 = PTV_5900. For proton therapy, 3 mm setup and 3%-range robustness are applied.

#### Dose evaluation

CTV_4000/CTV_5425 and CTV_5500/CTV_7000 coverage is evaluated according to standard of care (D98 > 94% in voxel-wise minimum plan for proton therapy, and D98 > 95% of the PTVs for photon therapy [29]). No more than 2% of the CTV_5500 excluding the GTV_5900 should receive more than 58.85 Gy (107% of 55 Gy).

The focal HYDRA boost to the GTV_5900 is delivered with a mean dose of 59 Gy and a maximum dose of 63.13 Gy (107% of 59 Gy). No more than 2% of the GTV_5900 should receive more than 63.13 Gy. In case of proton therapy, the GTV_5900 was evaluated nominally (i.e. not on voxel-wise minimum or maximum scenarios).

### Safety and predefined safety interim analyses

For each cohort (photon therapy and proton therapy), safety will be assessed by an independent Data Safety Monitoring Board (DSMB) with a predefined safety interim analysis, which occurs when ten patients treated with HYDRA have reached six months of follow-up. Any radiotherapy-related grade 3–4 toxicity ≥6 months after radiotherapy will be scored as a dose-limiting toxicity (DLT). Of note, late toxicity can occur even years after radiotherapy. Pragmatically, we considered ongoing/persistent severe acute toxicity ≥6 months after treatment and/or early onset late toxicity already within 6 months after treatment as good surrogates for (very) late toxicity and for considerations regarding trial continuation. As described in the study protocol [25], the DSMB will be consulted on whether to terminate the study (≥4 DLTs), continue inclusion according to initial inclusion criteria (2–3 DLTs) or expand the inclusion criteria with laryngeal carcinoma (≤1 DLT).

### Outcomes

In the current paper, we report on the predefined HYDRA-photon safety interim analysis. Toxicity was scored by the radiation oncologist according to CTCAE v5 at baseline, weekly during radiotherapy, and at planned follow-up visits at 2, 6, and 12 weeks; then every 3 months for a year, every 4 months up to 2 years, and every six months thereafter. Follow-up duration is calculated from the start of the first radiotherapy fraction.

Second, for all patients who were treated with the HYDRA schedule until the predefined interim analysis, we perform an intra-patient dosimetric comparison of the 20 fraction HYDRA plan and the clinically accepted 35 fraction conventional treatment plan (used for model-based plan comparison, as described earlier; see Fig. 1B). The comparison includes the dose to the target volumes and the dose received by relevant organs at risk (OARs).

## Results

The first patient was included in December 2022. In March 2024, ten patients treated with HYDRA-photons had reached six months follow-up, leading to the predefined safety interim analysis. In that period, a total of 15 patients were included in the HYDRA-photon arm and 5 patients in the HYDRA-proton arm.

### Planned interim analysis of HYDRA photon therapy

For the planned interim analysis of HYDRA photon therapy, per protocol all ten HYDRA-photon patients with at least six months follow-up were evaluated (median follow-up of 10.6 months, range 7.6–14.7 months). All patients were able to complete treatment as planned. Baseline clinical characteristics are provided in Supplementary Table 1.

A direct, detailed comparison of toxicity and outcomes between HYDRA and standard of care treatment is outside the scope of this preliminary evaluation and will be reported when the trial is completed. Here, we report on the planned interim analysis only.

For the safety evaluation in the planned interim analysis, we saw no tube feeding dependence or opioid use beyond 3 months after treatment. One patient had complicated wound healing following tooth extraction prior to the start of radiotherapy. This patient smoked until the start of radiotherapy. The first signs of osteonecrosis occurred approximately one month after radiotherapy, which led to a fracture of the mandible four months later. Dosimetrically, the maximum EQD2 dose (α/β = 3 Gy/0.85 Gy) to the mandible in the HYDRA plan was 67.7/75.3 Gy, compared to 73.2/73.9 Gy in the clinically accepted conventionally fractionated plan. The independent DSMB concluded that the delayed wound healing was negatively affected by the radiotherapy, but presumably not related to hypofractionation. There were no other late grade 3−5 toxicities following HYDRA.

As described in the trial protocol [25], the decision for trial continuation was based on the number of patients who experienced DLTs. Based on these results, the DSMB concluded that there was one DLT (osteoradionecrosis of mandible), which is within the predefined DLT limits. The trial could therefore be continued, and laryngeal carcinomas are now also amenable to inclusion in the photon cohort.

### Dosimetric treatment plan comparison

Intra-patient plan comparison was performed for all patients included up to the interim analysis. For one patient treated with HYDRA-photons, intra-patient dosimetric comparison between the HYDRA and conventional treatment plan was not possible due to minor changes in the target volumes. Baseline tumour characteristics of all other patients in both HYDRA arms used for the intra-patient plan comparison are provided in Table 1.

**Table 1:** Tumour characteristics of all patients used for the intra-patient plan comparison of HYDRA versus conventional fractionated treatment plans.

	HYDRA-photons	HYDRA-protons
Number of patients	14	5
**Tumour site**		
Oropharynx	10	5
p16 positive	6	5
p16 negative	2	0
p16 unknown	2	0
Hypopharynx	4	0
**T-stage**		
1	4	0
2	7	1
3	2	2
4	1	2
**N-stage**		
0	3	2
1	5	2
2	4	1
3	2	0
**Stage (AJCC 8th edition)**		
I	4	1
II	4	2
III	2	2
IV	4	0
**Mean target volume, cc (range)**		
GTVp	16.6 (3.3–81.2)	23.6 (15.5–37.6)
GTVn (total volume)	22.4 (11.4–44.9)	19.1 (10.2–27.9)
GTV_5900	13.7 (0.4–46.7)	14.3 (5.9–20.8)
CTV_5500	79.6 (15.2–161.3)	91.8 (41.1–132.0)

**Fig. 2:**
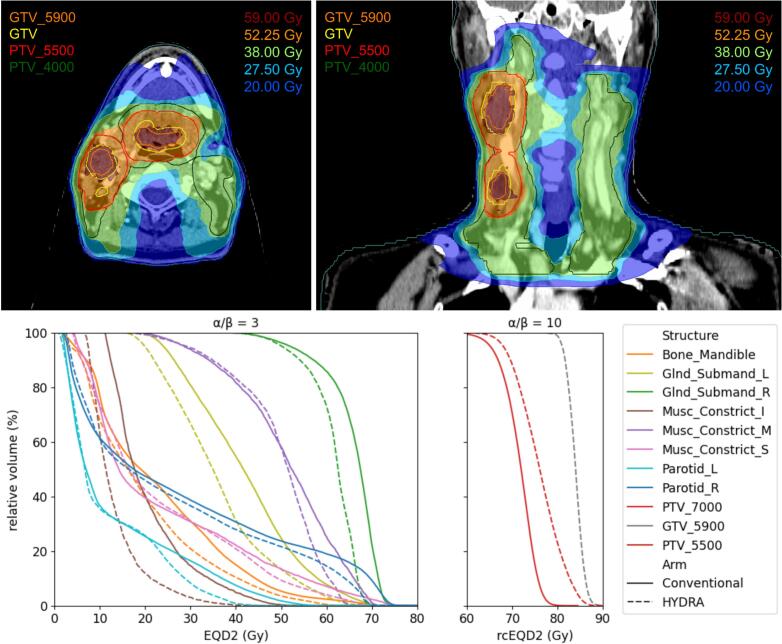
HYDRA treatment plan (top panels) and dose-volume histograms (DVHs) (bottom panels) for a patient with a cT2N1M0 oropharyngeal carcinoma. The dose to relevant organs at risk (OARs) are in equivalent dose in 2 Gy fractions (EQD2) using an α/β-value of 3 Gy. As described previously, the HYDRA CTV_5500 dose is partly based on new radiobiological modelling by Shuryak et al. [20], [21] suggesting that hypofractionated radiotherapy may result in higher tumour control by more efficiently targeting accelerated repopulation. In the bottom right panel, we therefore converted the dose to the target volumes into a repopulation-corrected EQD2 (rcEQD2) using the radiobiological parameters from Shuryak et al. (see appendix).

For illustrational purposes, Fig. 2 shows a representative example of a HYDRA-photon treatment plan with a focal boost to the centre of the GTVp and GTVn, as well as the doses to relevant target volumes and organs at risk (OARs) in equivalent dose in 2 Gy fractions (EQD2).

In all patients, target volume coverage was adequate (D98 > 95% for photons and D98 > 94% for protons) and similar between HYDRA and SOC. The mean dose to the CTV_5500 was 56.8 Gy (55.8–57.7 Gy) and was similar for HYDRA protons and HYDRA photons (see Table 2). All patients received a focal boost to the GTVp and (if there was a remaining volume after 3 mm contraction) to the GTVn. The mean dose to the GTV_5900 was 59.6 Gy (59.0–60.1 Gy) which was similar for HYDRA protons and HYDRA photons (see Table 2). In all patients, at most 2% of the GTV_5900 received 107% of the boost dose (<63.13 Gy). The D2% to the CTV_5500 was on average 60.6 Gy (59.3–61.5 Gy) (see Table 2).

**Table 2:** Mean dose to the target volumes and organs at risk per treatment plan. The dosimetric differences are presented separately for the HYDRA-photon arm (*n* = 14) and HYDRA-proton arm (*n* = 5). PCM: Pharyngeal Constrictor Muscle.

	Plan	HYDRA-photon arm (*n* = 14)	HYDRA-proton arm (*n* = 5)
*Mean physical dose, Gy (range)*			
CTV_5500/7000, Dmean	HYDRA	57.0 (55.8–57.7)	56.3 (56.1–56.5)
	SOC	71.6 (71.1–71.9)	71.1 (70.9–71.5)
GTV_5900, Dmean	HYDRA	59.7 (59.0–60.1)	59.4 (59.1–59.7)
	SOC	—	—
CTV_5500/7000, D2%	HYDRA	60.6 (59.3–61.5)	60.7 (60.2–61.0)
	SOC	74.0 (73.5–74.3)	73.9 (73.2–75.1)
Mean biological dose in rcEQD2*, Gy (range)			
CTV_5500/7000, Dmean	HYDRA	76.6 (73.9–78.2)	75.0 (74.6–75.5)
	SOC	73.2 (72.2–73.9)	72.2 (71.9–73.0)
GTV_5900, Dmean	HYDRA	82.7 (81.3–83.8)	82.1 (81.4–82.9)
	SOC	—	—
CTV_5500/7000, D2%	HYDRA	85.0 (82.0–87.0)	85.2 (83.9–86.0)
	SOC	78.2 (77.2–78.8)	78.0 (76.6–80.4)
*Mean volume receiving 70 Gy (EQD2 using α/β = 3 Gy), cc (range)*			
V70Gy	HYDRA	12.6 (0.6–38.6)	9.5 (3.7–13.6)
	SOC	88.8 (12.6–168.1)	110.3 (51.1–167.7)
*Mean biological dose in EQD2 using α/β = 3 Gy, Gy (range)*			
Ipsilateral parotid, Dmean	HYDRA	16.2 (2.4–57.1)	9.4 (6.5–13.1)
	SOC	20.0 (3.5–62.0)	11.6 (5.9–15.9)
Contralateral parotid, Dmean	HYDRA	10.5 (2.9–15.3)	5.9 (2.7–13.4)
	SOC	11.9 (3.3–15.2)	8.3 (4.2–18.4)
Ipsilateral submandibular gland, Dmean	HYDRA	52.6 (27.2–64.8)	45.8 (26.7–59.6)
	SOC	56.7 (29.8–69.0)	50.3 (34.9–63.3)
Contralateral submandibular gland, Dmean	HYDRA	30.2 (18.6–40.9)	28.2 (15.6–57.8)
	SOC	34.7 (26.7–50.0)	33.1 (24.1–60.1)
Oral cavity, Dmean	HYDRA	19.3 (4.3–34.2)	20.9 (15.3–25.8)
	SOC	20.8 (9.3–35.6)	23.0 (17.6–27.9)
PCM inferior, Dmean	HYDRA	27.3 (7.8–67.2)	12.7 (5.2–23.4)
	SOC	29.6 (8.3–71.8)	16.1 (8.1–33.1)
PCM medius, Dmean	HYDRA	46.2 (21.7–64.0)	36.4 (8.1–63.1)
	SOC	49.1 (32.0–69.4)	40.8 (12.8–69.0)
PCM superior, Dmean	HYDRA	38.4 (13.0–58.2)	47.3 (28.1–60.6)
	SOC	38.8 (13.7–64.3)	52.4 (32.0–65.8)
Mandible, D0.03cc	HYDRA	57.9 (29.1–68.3)	63.8 (62.4–64.9)
	SOC	66.0 (34.4–75.6)	73.2 (71.6–74.7)
Mandible, D30%	HYDRA	20.2 (5.1–32.8)	14.2 (5.4–21.5)
	SOC	24.6 (11.4–38.5)	17.4 (9.5–24.2)
*Mean biological dose in EQD2 using α/β = 0.85 Gy, Gy (range)*			
Mandible, D0.03cc	HYDRA	62.9 (27.2–76.1)	70.2 (68.4–71.7)
	SOC	65.5 (29.2–76.8)	73.9 (72.0–75.7)
Mandible, D30%	HYDRA	17.6 (3.2–31.4)	11.5 (3.5–18.6)
	SOC	19.6 (7.6–33.6)	12.9 (6.1–19.0)

* The repopulation-corrected EQD2 (rcEQD2) is an extension of the EQD2 which accounts for the effects of repopulation and overall treatment time on the efficacy, according to the radiobiological modelling of Shuryak et al. [20], [21]. The calculation is described in the supplementary methods (see appendix).

**Fig. 3:**
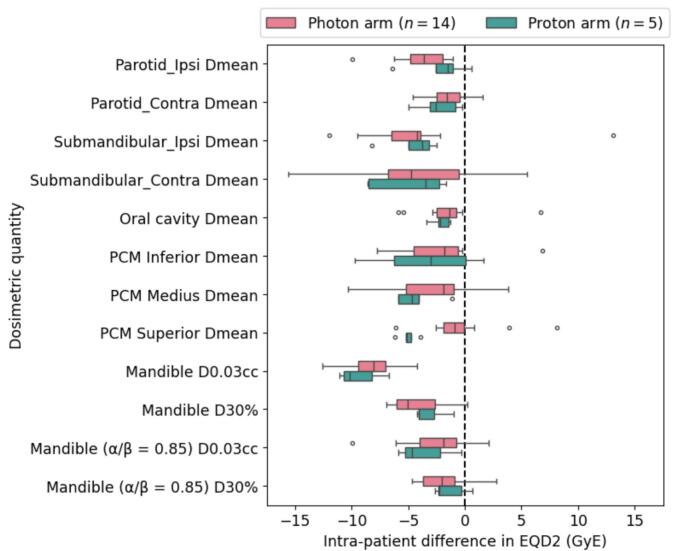
Intra-patient dosimetric comparison of HYDRA treatment plan versus standard of care treatment plan shows dose reductions in all organs at risk in the HYDRA treatment (in EQD2 using α/β = 3 Gy, unless otherwise specified). Coverage of target volumes was adequate and similar between both treatment plans (D98 > 95% for photons, D98 > 94% for protons) but cannot be compared in terms of EQD2 due to accelerated repopulation. Mean doses to target volumes and organs at risk per treatment plan are provided in Table 2. The dosimetric differences are presented separately for the HYDRA-photon arm (*n* = 14) and HYDRA-proton arm (*n* = 5).

The HYDRA focal boost of 59 Gy within the tumour centre is isotoxic to 70 Gy in the conventionally fractionated plan regarding late toxicity, considering an α/β-value of 3 Gy. The mean volume receiving an EQD2 of 70 Gy (α/β = 3 Gy) was on average 89% smaller with HYDRA versus conventional fractionation (11.7 cc vs 94.5 cc, *p*-value < 0.001, see Table 2). Compared to standard of care, with HYDRA there is an average reduction in the mean dose to organs at risk (OARs) of 0.4–4.5 Gy EQD2 for HYDRA-photons and 2.1–5.0 GyE for HYDRA-protons (see Fig. 3 for the average EQD2 reductions, the actual doses per treatment plan are provided in Table 2).

Analysis of dosimetric quantities for the mandible (in line with [30], [31]) using an α/β-value of 3 Gy and 0.85 Gy [32] shows that with HYDRA, the maximum dose (D0.03cc) and D30% are both reduced compared to the conventional plans. In the HYDRA plans, the mandible also receives a lower V70Gy (V55Gy for 20 fractions using α/β = 0.85 Gy), with a mean reduction of 0.15 cc (range −0.45 to 1.56 cc) for HYDRA-photons, and 0.35cc (range 0 to 0.64 cc) for HYDRA-protons.

## Discussion

In this paper, we present the first planned interim analysis for the HYDRA trial, which consists of two parallel phase I trials evaluating the safety of hypofractionated, dose-redistributed radiotherapy for HNSCC. Next, we perform an intra-patient dosimetric comparison of HYDRA vs standard of care. In summary, after a median follow-up of 10.6 months, the planned interim safety analysis for HYDRA-photon therapy showed one dose limiting toxicity, which is within the predefined DLT limits. So far, we have observed clinical feasibility of the HYDRA radiotherapy scheme, allowing further inclusion of patients in our study and expansion of the inclusion to laryngeal carcinoma patients. The dosimetric comparison demonstrated that the HYDRA schedule results in a lower EQD2 dose to all organs at risk, while delivering an inhomogeneous boost to the primary tumour and involved lymph nodes.

Hypofractionation for HNSCC patients could result in a reduced treatment burden for patients, while maintaining acceptable late toxicity and comparable efficacy. Additionally, with the combination of hypofractionation and a reduced elective lymph node dose, it may be possible to spare the immune system during radiotherapy. HYDRA is randomized with SOC to compare the immune effects of both fractionation schedules by longitudinal immune profiling of peripheral blood. The analysis of immune effects will be performed upon trial completion.

The COVID-19 pandemic has accelerated the use of hypofractionated regimens, leading to renewed evaluation of evidence for hypofractionated treatments [33]. Rising health care costs, growing personnel shortages and increased awareness of negative environmental effects necessitate further exploration of hypofractionated regimens to maintain future accessibility and sustainability of our health care system.

To our knowledge, this is the first study to assess hypofractionated definitive radiotherapy for HNSCC combining a focal boost to the GTV centre with a reduced elective lymph node dose. Although growing evidence supports lowering elective doses [16], [17], this has not been combined with hypofractionation. A Brazilian phase I trial using 20 × 2.75 Gy with concurrent cisplatin reported acute toxicity comparable to standard chemoradiotherapy, with limited long-term feeding-tube dependence [23]. Subgroup analyses from the PET-NECK study similarly found comparable locoregional control, survival, and quality of life after 20 × 2.75 Gy versus standard of care fractionation [24]. The large randomized HYPNO trial further demonstrated non-inferiority of this 20-fraction regimen for 3-year locoregional control and severe late toxicity [34]. These data were presented at ASTRO 2023, but final publication is pending. Similar results have been found in retrospective studies investigating schedules of 25 fractions up to a total dose of 60 Gy [35], 62.5 Gy [36] and 64 Gy [37].

The HYDRA scheme is mainly based on the radiobiological modelling of Shuryak et al. [20], [21], who suggest a dose of 18 × 3 Gy as the optimal balance between efficacy and late toxicity, which is approximately equivalent to 20 × 2.75 Gy to the CTV in our study. With our focal boost of 20 × 2.95 Gy to the GTV minus 3 mm, we aim to further secure tumour control by a focal boost within the tumour centre along with a steep dose fall-off within the PTV, so that neighbouring OARs are not compromised (concept adapted from the ARTFORCE trial [19]). The primary goal of the dosimetric analysis is to demonstrate that HYDRA does not result in increased OAR dose (and thus potentially more late toxicity). In contrast, we show that OARs consistently receive a lower dose with HYDRA (photons: 0.4 to 4.5 Gy EQD2; protons: 2.1 to 5.0 GyE EQD2). Evidence indicates that incremental reductions in OAR doses are associated with improvements in late toxicity [38], [39]. Whether the relatively small OAR dose reductions in our trial lead to less late toxicity will be evaluated at trial completion.

Our study is subject to some limitations. We aim to demonstrate the applicability of HYDRA as a universal treatment for all HNSCC patients, regardless of tumour site, size, HPV status, nodal involvement or use of concurrent systemic therapy. Nevertheless, we have predominantly recruited HPV-positive patients and there are currently no HPV-negative patients in the proton therapy cohort. Also, toxicity is different between tumour sites and with use of concurrent systemic therapy, complicating the interpretation of toxicity data. Secondly, accrual in the proton cohort is slow compared to the photon cohort due to model-based selection and logistical challenges, unrelated to the study protocol. Further inclusion of these patients should give a better understanding of the effects of hypofractionated proton therapy. Lastly, while our interim results look promising, the minimum follow-up of 6 months is short for adequate evaluation of late toxicity. Longer follow-up and more patients are needed for further evaluation. By the design of our trial, we can enrol up to 40 patients in each photon therapy arm, and up to 25 patients in each proton therapy arm. Trial continuation will depend on the outcome of the pre-specified HYDRA-proton interim analysis.

In conclusion, the HYDRA schedule consists of only 20 instead of the conventional 35 fractions. For HYDRA-photons, the predefined interim analysis demonstrated clinical feasibility, with late toxicity within predefined limits and in line with standard of care treatment. Following this predefined interim analysis, laryngeal carcinoma patients are now also included in the photon cohort. The intra-patient dosimetric comparison confirmed that HYDRA achieves an inhomogeneous focal boost to the primary tumour and involved lymph nodes, while resulting in a lower EQD2 dose to the organs at risk. As accrual is ongoing, the effects of HYDRA on toxicity and the immune system will be reported once mature results become available.

## Funding statement

This work was supported by the Daniel den Hoed Award and the Dutch Cancer Society (KWF). The funding sources had no role in study design, data collection, analysis, interpretation, or writing of the article.

## Declaration of interests

The authors declare that they have no known competing financial interests or personal relationships that could have appeared to influence the work reported in this paper.

## CRediT authorship contribution statement

**Pascal A. Gunsch:** Conceptualization, Data curation, Formal analysis, Methodology, Project administration, Resources, Writing – original draft, Writing – review & editing. **Michiel Kroesen:** Data curation, Resources, Writing – review & editing. **Reno Debets:** Data curation, Writing – review & editing. **Stijn Keereweer:** Data curation, Resources, Writing – review & editing. **Esther van Meerten:** Data curation, Resources, Writing – review & editing. **Jaap Zindler:** Data curation, Resources, Writing – review & editing. **Erik van Werkhoven:** Formal analysis, Writing – review & editing. **Mischa Hoogeman:** Conceptualization, Formal analysis, Supervision, Writing – review & editing. **Gerda M. Verduijn:** Data curation, Resources, Writing – review & editing. **Remi A. Nout:** Conceptualization, Methodology, Project administration, Supervision, Writing – review & editing. **Joris B.W. Elbers:** Conceptualization, Methodology, Project administration, Resources, Supervision, Writing – original draft, Writing – review & editing.
