# Supplementary material for: HYpofractionated, dose-redistributed RAdiotherapy (HYDRA) versus conventional radiotherapy for head and neck cancer: planned interim analysis and dosimetric comparison from the phase I HYDRA trial
Source: Clin Transl Radiat Oncol. 2026 Jan 25;58:101113. doi: 10.1016/j.ctro.2026.101113 (PMC12890858; doi:10.1016/j.ctro.2026.101113)
Supplement: Supplementary Data 1 [file mmc1.docx]

## Supplementary data

*Table S1 Patient characteristics analysed in the interim analysis (HYDRA-photons, n = 10)*

|  | HYDRA-photons |
| --- | --- |
| Number of patients | 10 |
| Mean age, years (range) | 62 (38−74) |
| **Sex** |  |
| Male | 7 |
| Female | 3 |
| Other | 0 |
| **Smoking status** |  |
| Current | 6 |
| Former (>3 months ago) | 1 |
| Never | 3 |
| **Pack years (in ever smokers)** |  |
| >10 pack years | 7 |
| *≤*10 pack years | 0 |
| **Alcohol abuse** |  |
| Current | 1 |
| Former (>3 months ago) | 2 |
| Never | 7 |
| **Tumour site** |  |
| Oropharynx | 8 |
| p16 positive | 5 |
| p16 negative | 2 |
| p16 unknown | 1 |
| Hypopharynx | 2 |
| **T-stage** |  |
| 1 | 4 |
| 2 | 5 |
| 3 | 0 |
| 4 | 1 |
| **N-stage** |  |
| 0 | 3 |
| 1 | 3 |
| 2 | 2 |
| 3 | 2 |
| **Stage (AJCC 8th edition)** |  |
| I | 3 |
| II | 4 |
| III | 1 |
| IV | 2 |
| **Radiosensitiser** |  |
| Cisplatin/Carboplatin | 5 |
| Cetuximab | 0 |
| None | 5 |
