# Supplementary material for: HYpofractionated, dose-redistributed RAdiotherapy (HYDRA) versus conventional radiotherapy for head and neck cancer: planned interim analysis and dosimetric comparison from the phase I HYDRA trial
Source: Clin Transl Radiat Oncol. 2026 Jan 25;58:101113. doi: 10.1016/j.ctro.2026.101113 (PMC12890858; doi:10.1016/j.ctro.2026.101113)
Supplement: Supplementary Data 2 [file mmc2.pdf]

Appendix: Calculation of repopulation-corrected EQD2

1. Introduction

In radiobiology, the effects of altered fractionation on clinical outcomes is most commonly modelled using the linear-quadratic (LQ) model [1]. The radiobiological models described by the LQ model assume that a total dose D is given in n fractions, each with equal fraction dose d, so that $D = nd$. Additionally, the time between fractions is long enough for sufficient recovery of the tissues between fractions. In this case, the cell survival (assumed to be correlated with clinical effects) is given by:

$$S = \exp\left(-\alpha nd - \beta nd^2\right) \tag{1}$$

In this simple model, two interrelated quantities are often used to compare different fractionation schedules:

(1) Biologically effective dose (BED). Though the interpretation of the BED in terms of clinically relevant quantities is not straightforward, it can be directly derived from the expression for the cell survival S using:

$$\mathrm{BED} = \frac{-\ln S}{\alpha} \tag{2}$$

Substituting equation 1:

$$\mathrm{BED} = nd\left(1 + \frac{d}{\alpha/\beta}\right) \tag{3}$$

Thus, the BED is the product of the total dose ($D = nd$) and a modifying factor called the relative effectiveness (RE):

$$\mathrm{RE} = 1 + \frac{d}{\alpha/\beta} \tag{4}$$

(2) Equivalent dose in 2 Gy fractions (EQD2). The EQD2 can be interpreted as the dose, given in constant fractions of 2 Gy, that yields the same clinical effect as the reference dose which is given with a different fraction dose.

$$\mathrm{EQD2} = D_{\mathrm{ref}} \cdot \frac{\mathrm{RE}_{\mathrm{ref}}}{\mathrm{RE}_{2\,\mathrm{Gy}}} = \frac{\mathrm{BED}_{\mathrm{ref}}}{\mathrm{RE}_{2\,\mathrm{Gy}}} \tag{5}$$

$$\mathrm{EQD2} = D_{\mathrm{ref}} \cdot \frac{d_{\mathrm{ref}} + \alpha/\beta}{2 + \alpha/\beta} \tag{6}$$

2. Accounting for traditional accelerated repopulation

The traditional model for accelerated repopulation as introduced by Withers et al. [2] includes a delay period without repopulation, followed by a period of constant repopulation. To extend the LQ model to account for accelerated repopulation (AR), the kick-off time T_k and repopulation rate γ are introduced:

$$S = \exp\left(-\alpha nd - \beta nd^2 + \gamma(T - T_k)\right) \tag{7}$$

Note that this equation only applies if $T \geq T_k$.

Substituting this expression into equation 1:

$$\mathrm{BED} = D \cdot \mathrm{RE} - K(T - T_k) \tag{8}$$

where $K = \gamma/\alpha$.

2.1. *Calculation of the repopulation-corrected EQD2*

The inclusion of a time factor in the BED calculation complicates the calculation of a traditional EQD2, which is independent of the overall treatment time.

We will therefore introduce the repopulation-corrected EQD2 (rcEQD2), which is the total dose in fractions of 2 Gy, with a similar BED to the reference schedule. Thus, the following equation must be satisfied:

$$\mathrm{BED}_{2\,\mathrm{Gy}} = \mathrm{rcEQD2} \cdot \mathrm{RE}_{2\,\mathrm{Gy}} - K\left(T_{2\,\mathrm{Gy}} - T_k\right) = \mathrm{BED}_{\mathrm{ref}} \tag{9}$$

Here, $T_{2\,\mathrm{Gy}}$ is the time it takes to complete the treatment in 2 fractions. To find $T_{2\,\mathrm{Gy}}$, we will use an approach comparable to that of Dale *et al.* [3].

The total number of fractions n is given by $n = \mathrm{rcEQD2}/2$. Let n_w be the number of fractions in a single week, where the fractions are given for 5 days and there is a rest period of 2 days. Let f be the mean inter-fraction interval, given by $f = 7/n_w$.

If $n > n_w$, the overall treatment time in 2 Gy fractions can be calculated with an accuracy of one to two days by

$$T_{2\,\mathrm{Gy}} = fn - 2 = f \cdot (\mathrm{rcEQD2}/2) - 2 \tag{10}$$

Then, substituting equation 10 into equation 9:

$$\mathrm{BED}_{\mathrm{ref}} = \mathrm{rcEQD2} \cdot \mathrm{RE}_{2\,\mathrm{Gy}} - K((f/2) \cdot \mathrm{rcEQD2} - 2 - T_k) \tag{11}$$

Isolating rcEQD2, this results in:

$$\mathrm{rcEQD2} = \frac{1}{\mathrm{RE}_{2\,\mathrm{Gy}} - \frac{fK}{2}}\left[\mathrm{BED}_{\mathrm{ref}} - K(2 + T_k)\right] \tag{12}$$

3. Accounting for repopulation using the dose-independent repopulation (DIR) model of Shuryak et al.

The dose-independent repopulation (DIR) model of Shuryak et al. [4, 5] is an extension of the Withers model, additionally including continuous tumor repopulation that occurs during treatment ('regular' instead of accelerated tumor growth).

In the DIR model, the survival function is given by:

$$S_{\mathrm{DIR}} = \exp\left(-\alpha nd - \beta nd^2 + \gamma(T - T_k) + gT\right) \tag{13}$$

Substituting this into the expression for the BED:

$$\mathrm{BED_{DIR}} = D \cdot \mathrm{RE} - K(T - T_\mathrm{k}) - GT \tag{14}$$

where $K = \gamma/\alpha$ and $G = g/\alpha$.

3.1. Calculation of the repopulation-corrected EQD2

Similar to the discussion above, the rcEQD based on the DIR model is calculated based on the $\mathrm{BED_{2\,Gy}}$:

$$\mathrm{BED_{2\,Gy}} = \mathrm{rcEQD2} \cdot \mathrm{RE_{2\,Gy}} - K(f/2 \cdot \mathrm{rcEQD2} - 2 - T_\mathrm{k}) - G(f/2 \cdot \mathrm{rcEQD2} - 2) \tag{15}$$

$$= \left(\mathrm{RE_{2\,Gy}} - \frac{Kf}{2} - \frac{Gf}{2}\right)\mathrm{rcEQD2} + K(2 + T_\mathrm{k}) + 2G \tag{16}$$

The rcEQD2 can again be calculated from the equivalence of the $\mathrm{BED_{ref}}$ and $\mathrm{BED_{2\,Gy}}$:

$$\mathrm{BED_{ref}} = \left(\mathrm{RE_{2\,Gy}} - \frac{Kf}{2} - \frac{Gf}{2}\right)\mathrm{rcEQD2} + K(2 + T_\mathrm{k}) + 2G \tag{17}$$

Isolating rcEQD2, this results in:

$$\mathrm{rcEQD2} = \frac{1}{\mathrm{RE_{2\,Gy}} - \frac{Kf}{2} - \frac{Gf}{2}}\left[\mathrm{BED_{ref}} - K(2 + T_k) - 2G\right] \tag{18}$$

4. Conclusions

In this appendix, we have provided two formulae to calculate the repopulation-corrected EQD2 (rcEQD2) using the traditional accelerated repopulation model as well as the dose-independen repopulation model of Shuryak et al. These provide an intuitive way to compare different fractionation schemes in the presence of accelerated repopulation.

References

[1] S. J. McMahon, *The linear quadratic model: usage, interpretation and challenges*, Physics in Medicine & Biology **64** (2018) 01TR01.

[2] H. Withers, J. Taylor and B. Maciejewski, *The hazard of accelerated tumor clonogen repopulation during radiotherapy*, Acta oncologica **27** (1988) 131.

[3] R. Dale, G. Plataniotis and B. Jones, *A generalised method for calculating repopulation-corrected tumour eqd2 values in a wide range of clinical situations, including interrupted treatments*, Physica Medica **118** (2024) 103294.

[4] I. Shuryak, E. J. Hall and D. J. Brenner, *Dose dependence of accelerated repopulation in head and neck cancer: Supporting evidence and clinical implications*, Radiotherapy and Oncology **127** (2018) 20.

[5] I. Shuryak, E. J. Hall and D. J. Brenner, *Optimized hypofractionation can markedly improve tumor control and decrease late effects for head and neck cancer*, International Journal of Radiation Oncology* Biology* Physics **104** (2019) 272.
